# An era of hyper-resolution land surface models: truth or dare?

**DOI:** 10.1093/nsr/nwaf285

**Published:** 2025-07-28

**Authors:** Tingting Wu, Yuan He, Xiaofan Yang, Yongjiu Dai

**Affiliations:** Guangdong Provincial Observation and Research Station for Coupled Human and Natural Systems in Land-ocean Interaction Zone, Beijing Normal University at Zhuhai, China; State Key Laboratory of Earth Surface Processes and Disaster Risk Reduction, Faculty of Geographical Science, Beijing Normal University, China; Guangdong Provincial Observation and Research Station for Coupled Human and Natural Systems in Land-ocean Interaction Zone, Beijing Normal University at Zhuhai, China; State Key Laboratory of Earth Surface Processes and Disaster Risk Reduction, Faculty of Geographical Science, Beijing Normal University, China; Guangdong Provincial Observation and Research Station for Coupled Human and Natural Systems in Land-ocean Interaction Zone, Beijing Normal University at Zhuhai, China; State Key Laboratory of Earth Surface Processes and Disaster Risk Reduction, Faculty of Geographical Science, Beijing Normal University, China; School of Atmospheric Sciences, Sun Yat-sen University, China

## Abstract

Hyper-resolution land surface models (hLSMs) simulate land processes at kilometer and even 100-meter scales by integrating biogeophysical, biogeochemical, and human dynamics, enhancing realism. However, substantially improving their accuracy necessitates parallel progress in parameterizations, data, computational capabilities, and model evaluation.

Land surface models (LSMs) are vital for simulating water, energy and carbon fluxes between land surface and atmosphere [1]. To address challenges in climate science, ecology and society, LSMs now incorporate previously oversimplified processes spanning biogeophysics, biogeochemistry, ecosystem dynamics and human influences (Fig. 1a and b). Advances in data, schemes and computing enable finer spatial and temporal resolutions, leading to hyper-resolution LSMs (hLSMs) operating at approximately 1 km globally and 100 m continentally [2]. This shift aims for more realistic process representation and improved accuracy by resolving finer scales of motion. However, higher resolution and complexity do not guarantee better accuracy. Poorly tuned parameterizations or weakly integrated modules can limit model performance [3]. Therefore, advancing hLSMs requires improvement of parameterization schemes, bridging scale gaps between achievable resolution and available data, and enhancing computational power and numerical methods [4]. This perspective discusses key necessities, challenges, opportunities and vision for hLSMs (Fig. 1c).

**Figure 1.**
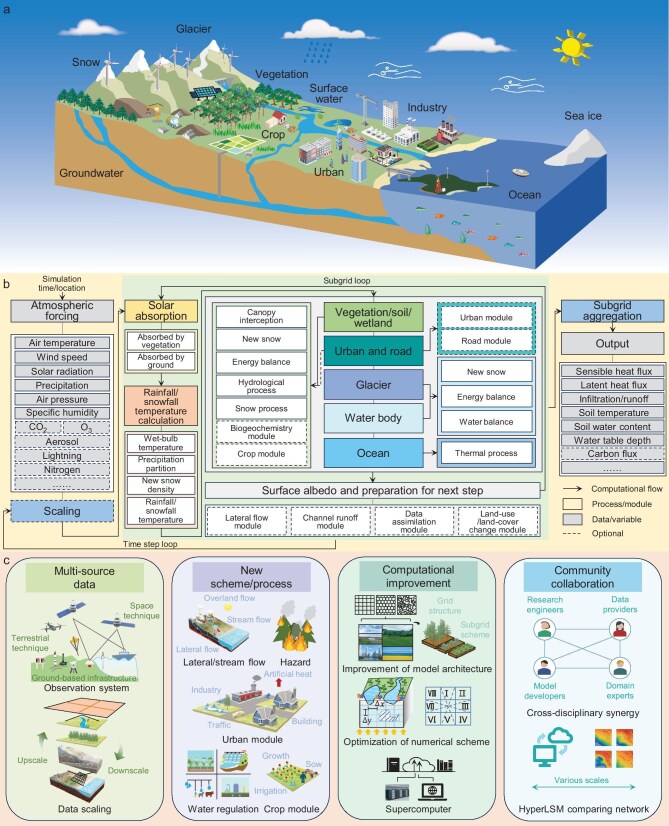
(a) Conceptual schematic of a land surface model. (b) Computing framework of a land surface model, illustrated with CoLM 2024 as an example (modified from https://github.com/CoLM-SYSU/CoLM-doc/). (c) Major advances towards hyper-resolution LSMs.

The hLSMs are necessary to enhance representation of land surface characteristics, hydrologic processes and human activities. (i) Traditional LSMs empirically approximate fine-scale land surface characteristics (e.g. topography, soil texture and vegetation), representing changes through parameters like topographic index, soil hydrothermal parameters and albedo [5]. However, coarse spatial resolution limits the capture of fine-scale surface heterogeneity. The hLSMs overcome this by enhancing spatial resolution, incorporating detailed inputs and enabling subgrid parameterizations. These improvements enhance representation of real-world processes across scales and simulation accuracy. For example, slope effects on hydrology are captured at ≤100 m scales using high-resolution topography and soil data [5], ensuring key variables better align with real-world characteristics. (ii) Hydrologic processes in LSMs depend on topography and soil texture. Higher-resolution terrain data thus enhance their representation in hLSMs, overcoming the limitations of coarse-scale models. Recent developments in hLSMs have integrated more processes, refined model structures and improved numerical solutions. Key improvements include revised solutions for the Richards’ equation [6], better lateral flow representation [7] and integrated irrigation schemes [3]. These advances are critical for modeling hydrologic processes in complex landscapes and for applications in extreme event prediction, water management and field-scale decisions. (iii) Human activities reflect the Anthropocene's shifts in land use, water utilization and vegetation patterns [8]. These changes are now represented in LSMs through physical processes. This aligns with global hydrological models (GHMs) but differs from integrated assessment models (IAMs) that link economic systems with simplified climate models. In hLSMs, activities such as agricultural water use, reservoir regulation and energy infrastructure development, e.g. large-scale photovoltaic (PV) roofs, are parameterized within urban, irrigation, reservoir and energy balance models [2,9,10], using data on urban cover, vegetation, water bodies and infrastructure characteristics (e.g. PV surface roughness length). For instance, by modulating surface energy balances, air temperature patterns and building cooling loads, PV systems highlight the trade-offs inherent in climate adaptation strategies, underscoring the need for integrated assessment in sustainable urban planning. Incorporating such processes enables hLSMs to capture coupled human–water–energy–ecosystem dynamics with greater realism. Future efforts may also benefit from targeted field experiments to validate and improve the representation of human activities in hLSMs.

Development of hLSMs faces challenges in high-resolution data scarcity, parameterization scheme inadequacy, computational constraints and intercomparison and benchmarking limitations. (i) High-resolution meteorological forcing and land surface datasets are crucial to advance LSMs to hLSMs. However, limited spatiotemporal coverage of observations complicates parameter calibration and model validation. Although remote sensing offers global-scale and spatially continuous data, its coarse temporal resolution and mismatch with *in situ* measurements impede representation of surface heterogeneity [11]. The core data challenge lies not only in increasing resolution, but also in aligning model and data scales, and in reconciling remote sensing data with ground-truth observations [4]. (ii) Traditional LSMs often simplify fine-scale processes by assuming they're negligible at coarse resolutions. However, in hLSMs, these simplifications break down and require scale-consistent or refined parameterization schemes [7]. For example, hLSMs incorporate urban hydrology by accounting for the impacts of impervious surfaces on heat and water fluxes [2]. Advancing high-resolution parameterizations also calls for new concepts or methods, such as eco-evolutionary optimality (EEO)-based schemes that enhance predictive accuracy with fewer parameters [12], root-zone-based representations that highlight its role in regulating water fluxes [13] and structural advances from big-leaf to big-tree models [14]. Moreover, parameterizations must reflect cross-module interactions. For instance, mismatches between detailed surface and simplified subsurface parameterizations require relaxed boundary conditions or advanced features like variable saturation flow and parallel processing [2]. (iii) The hLSMs pose significant computational challenges. For instance, a 1 km LSM can be 100 times more computationally demanding than a 10 km LSM [4]. This requires high-performance computing resources and optimized numerical schemes with advanced cyber infrastructure (e.g. OpenMP-CUDA parallelism) [15]. Furthermore, coupling hLSMs with coarser models adds complexity, requiring modular strategies that ensure scalability and resource efficiency [2]. Aligning model design with state-of-the-art software and hardware architectures is essential to overcome these hurdles. (iv) The intercomparisons of LSMs have progressed through efforts such as the Project for the Intercomparison of Land-surface Parameterization Schemes (PILPS) and the Global Land–Atmosphere Coupling Experiment (GLACE), which reveal model–observation gaps across scales. Benchmarking, started by the Global Land-Atmosphere System Study (GLASS) and developed into the Protocol for the Analysis of Land Surface (PALS) models, targets shared areas for improvement. However, differences in model complexity, limited high-resolution data and spin-up issues hinder effective validation. Fully coupled LSMs introduce added uncertainty through feedback among components. Building benchmarks to assess the entire coupled system remains a primary yet difficult objective. These issues are amplified in hLSMs, which are highly sensitive to uncertainties in input data and model parameters.

Potential solutions, including utilizing big data resources, advancing modeling techniques, leveraging high-performance computing and fostering community-level collaboration, have been proposed to enhance hLSMs. (i) Big data from field surveys, *in situ* observation networks and satellite remote sensing offer critical information on vegetation, topography, climate and soil. Systems like FLUXNET (a global network measuring carbon and energy fluxes), the International Soil Moisture Network (ISMN) and COsmic-ray Soil Moisture Observing System (COSMOS) provide detailed land-surface observations, while the Shuttle Radar Topography Mission (SRTM) and Advanced Spaceborne Thermal Emission and Reflection Radiometer (ASTER) offer high-resolution remote sensing data. Global datasets, including MERIT Hydro (a high-resolution global hydrography map) and GLobal HYdrogeology MaPS (GLHYMPS), improve representations of river networks and aquifer permeability. Beyond data collection, integrating big data through advanced technologies is crucial. For example, Hierarchical multi-scale clustering (HMC) groups high-dimensional data into hydrologically connected clusters (i.e. tiles), improving data utility and reducing computational demand [16]. Machine learning (ML)-based spatial downscaling bridges the resolution gap between satellite data and field applications [11]. This is especially important in mountainous or heterogeneous regions, where traditional data interpolation or fusion methods fall short. In these areas, integrating high-resolution land-atmosphere simulations with remote sensing can generate high-resolution forcing data [17]. Data assimilation further improves model accuracy by aligning simulations with observed data [1]. Altogether, the generation and use of high-resolution information significantly enhance understanding of the structure and function of hLSMs. (ii) Techniques for developing hLSMs focus on improved process representation and effective data utilization. Fast processes (e.g. photosynthesis) suit ML-based parameterizations due to ample data, while slower processes (e.g. carbon allocation) benefit from hybrid models combining ML and physical constraints [18]. This integration, such as embedding ML within models or using it to refine physical model parameters, enhances accuracy by utilizing data and supporting extrapolation. Moreover, advances enable scalable schemes through scale-dependent parameter estimation and closure parameterizations [19]. Coupling submodules also improves simulations, but requires computational cost–benefit analysis for added complexity [3]. Furthermore, flexible coupling in hLSMs balances efficiency and accuracy by loosely coupling weak-feedback processes and tightly coupling key modules. Introducing these advanced physical models or parametric representations improves performance of hLSMs. (iii) Advances in model structure, numerical algorithms, ML and computing power have improved the efficiency of hLSMs. To balance model complexity with computational efficiency, hLSMs can generate hydrological response units (HRUs) through multi-scale hierarchical clustering, which reduces system dimensionality [11]. Additionally, triangulated irregular network (TIN)-based modeling can capture complex terrain with minimal information [20]. In numerical algorithms, adaptive mesh refinement (AMR) further boosts efficiency by dynamically adjusting grid resolution [3]. ML can accelerate computationally expensive processes [18]. Moreover, advanced parallel computing techniques, along with cloud and supercomputing resources, further enhance simulation performance. (iv) Community-level collaboration among domain experts, model developers, engineers and data providers ensures the robustness of hLSMs through the exchange of data, techniques and process definitions. Specifically, open access to data and code promotes transparency and innovation, while partnerships between academia and policymakers support research and technology transfer. Synergy between scientific and technical experts enables the development of physically consistent coupling schemes that address model complexity while maintaining computational feasibility. Collaborative efforts are further strengthened by joint publications, shared platforms and working groups like HyperHydro (https://resolver.tudelft.nl/uuid:39dac170-b51e-49b2-94ea-5f0d9b565914), which advance model evaluation and usability through better interfaces and diagnostic tools. Finally, training programs are critical for promoting the adoption and application of hLSMs.

The hLSMs are not merely finer-grid versions of old models but are enriched with high-resolution data, customized parameterizations and additional physical processes that are absent from coarse-resolution LSMs. As complexity grows, so do computational demands and validation challenges. It is therefore essential to assess whether switching to hLSMs actually improves model performance. The focus should be on ‘hLSMs by necessity’, not ‘hLSMs by capability’. With continued advancements in data collection, modeling techniques and computational power, hLSMs are set to become the standard, providing globally consistent yet locally relevant insights. Ultimately, advancing this field requires coordinated efforts to integrate observations, models and theory across scientific communities.
